# Supplementary material for: Transcranial Magnetic Stimulation for the Treatment of Cocaine Addiction: A Systematic Review
Source: J Clin Med. 2021 Nov 28;10(23):5595. doi: 10.3390/jcm10235595 (PMC8658408; doi:10.3390/jcm10235595)
Supplement: Supplementary file 1 [file jcm-10-05595-s001.zip › jcm-1460098-supplementary.pdf]

Table S1. PRISMA Check-list.

Section and Topic	Item #	Checklist item	Location where item is reported
**TITLE**			
Title	1	Identify the report as a systematic review.	Page 1
**ABSTRACT**			
Abstract	2	See the PRISMA 2020 for Abstracts checklist.	Page 1
**INTRODUCTION**			
Rationale	3	Describe the rationale for the review in the context of existing knowledge.	Pages 1-3
Objectives	4	Provide an explicit statement of the objective(s) or question(s) the review addresses.	Page 3
**METHODS**			
Eligibility criteria	5	Specify the inclusion and exclusion criteria for the review and how studies were grouped for the syntheses.	Pages 3-4
Information sources	6	Specify all databases, registers, websites, organisations, reference lists and other sources searched or consulted to identify studies. Specify the date when each source was last searched or consulted.	Page 4
Search strategy	7	Present the full search strategies for all databases, registers and websites, including any filters and limits used.	Table 1<br>Additional file 2
Selection process	8	Specify the methods used to decide whether a study met the inclusion criteria of the review, including how many reviewers screened each record and each report retrieved, whether they worked independently, and if applicable, details of automation tools used in the process.	Page 4
Data collection process	9	Specify the methods used to collect data from reports, including how many reviewers collected data from each report, whether they worked independently, any processes for obtaining or confirming data from study investigators, and if applicable, details of automation tools used in the process.	Page 4
Data items	10a	List and define all outcomes for which data were sought. Specify whether all results that were compatible with each outcome domain in each study were sought (e.g. for all measures, time points, analyses), and if not, the methods used to decide which results to collect.	Page 4
	10b	List and define all other variables for which data were sought (e.g. participant and intervention characteristics, funding sources). Describe any assumptions made about any missing or unclear information.	Page 4
Study risk of bias assessment	11	Specify the methods used to assess risk of bias in the included studies, including details of the tool(s) used, how many reviewers assessed each study and whether they worked independently, and if applicable, details of automation tools used in the process.	Pages 4-5
Effect measures	12	Specify for each outcome the effect measure(s) (e.g. risk ratio, mean difference) used in the synthesis or presentation of results.	Not applicable

Section and Topic	Item #	Checklist item	Location where item is reported
Synthesis methods	13a	Describe the processes used to decide which studies were eligible for each synthesis (e.g. tabulating the study intervention characteristics and comparing against the planned groups for each synthesis (item #5)).	Not applicable
	13b	Describe any methods required to prepare the data for presentation or synthesis, such as handling of missing summary statistics, or data conversions.	Not applicable
	13c	Describe any methods used to tabulate or visually display results of individual studies and syntheses.	Not applicable
	13d	Describe any methods used to synthesize results and provide a rationale for the choice(s). If meta-analysis was performed, describe the model(s), method(s) to identify the presence and extent of statistical heterogeneity, and software package(s) used.	Not applicable
	13e	Describe any methods used to explore possible causes of heterogeneity among study results (e.g. subgroup analysis, meta-regression).	Not applicable
	13f	Describe any sensitivity analyses conducted to assess robustness of the synthesized results.	Not applicable
Reporting bias assessment	14	Describe any methods used to assess risk of bias due to missing results in a synthesis (arising from reporting biases).	Not applicable
Certainty assessment	15	Describe any methods used to assess certainty (or confidence) in the body of evidence for an outcome.	Not applicable
**RESULTS**			
Study selection	16a	Describe the results of the search and selection process, from the number of records identified in the search to the number of studies included in the review, ideally using a flow diagram.	Page 5
	16b	Cite studies that might appear to meet the inclusion criteria, but which were excluded, and explain why they were excluded.	Not applicable
Study characteristics	17	Cite each included study and present its characteristics.	Page 5<br>Table 2
Risk of bias in studies	18	Present assessments of risk of bias for each included study.	Pages 8-10, Figures 2-3; Additional files 3-4
Results of individual studies	19	For all outcomes, present, for each study: (a) summary statistics for each group (where appropriate) and (b) an effect estimate and its precision (e.g. confidence/credible interval), ideally using structured tables or plots.	Not applicable
Results of syntheses	20a	For each synthesis, briefly summarise the characteristics and risk of bias among contributing studies.	Pages 12-15

Section and Topic	Item #	Checklist item	Location where item is reported
	20b	Present results of all statistical syntheses conducted. If meta-analysis was done, present for each the summary estimate and its precision (e.g. confidence/credible interval) and measures of statistical heterogeneity. If comparing groups, describe the direction of the effect.	Not applicable
	20c	Present results of all investigations of possible causes of heterogeneity among study results.	Not applicable
	20d	Present results of all sensitivity analyses conducted to assess the robustness of the synthesized results.	Not applicable
Reporting biases	21	Present assessments of risk of bias due to missing results (arising from reporting biases) for each synthesis assessed.	Not applicable
Certainty of evidence	22	Present assessments of certainty (or confidence) in the body of evidence for each outcome assessed.	Not applicable
**DISCUSSION**			
Discussion	23a	Provide a general interpretation of the results in the context of other evidence.	Pages 15-17
	23b	Discuss any limitations of the evidence included in the review.	Page 17
	23c	Discuss any limitations of the review processes used.	Page 17
	23d	Discuss implications of the results for practice, policy, and future research.	Page 17
**OTHER INFORMATION**			
Registration and protocol	24a	Provide registration information for the review, including register name and registration number, or state that the review was not registered.	Page 3
	24b	Indicate where the review protocol can be accessed, or state that a protocol was not prepared.	Page 3
	24c	Describe and explain any amendments to information provided at registration or in the protocol.	Not applicable
Support	25	Describe sources of financial or non-financial support for the review, and the role of the funders or sponsors in the review.	Not reported
Competing interests	26	Declare any competing interests of review authors.	Page 17
Availability of data, code and other materials	27	Report which of the following are publicly available and where they can be found: template data collection forms; data extracted from included studies; data used for all analyses; analytic code; any other materials used in the review.	Not reported

From: Page MJ, McKenzie JE, Bossuyt PM, Boutron I, Hoffmann TC, Mulrow CD, et al. The PRISMA 2020 statement: an updated guideline for reporting systematic reviews. BMJ 2021;372:n71. doi: 10.1136/bmj.n71For more information, visit: http://www.prisma-statement.org/

Table S2. Search strategy.

**MEDLINE**		
1	Transcranial Magnetic Stimulation/	11631
2	(transcranial adj1 magnetic stimulation$).tw.	14512
3	((transcranial magnetic stimulation or tms) adj5 repetitive).tw.	4917
4	((transcranial magnetic stimulation or tms) adj5 rhythmic).tw.	41
5	(rtms or tms).tw.	15423
6	((Repetitive or "single pulse" or "paired pulse") adj1 "transcranial magnetic stimulation").tw.	5260
7	1 or 2 or 3 or 4 or 5 or 6	21895
8	Cocaine-Related Disorders/	8278
9	(cocaine* adj2 (abuse* or addict* or dependen* or disorder*)).ti,ab.	7622
10	8 or 9	12519
11	7 and 10	46

**EMBASE**		
1	'transcranial magnetic stimulation'/exp	24,787
2	(transcranial NEAR/1 'magnetic stimulation*'): ti, ab, de	26,601
3	(('transcranial magnetic stimulation' OR tms) NEAR/5 repetitive): ti, ab, de	7,908
4	(('transcranial magnetic stimulation' OR tms) NEAR/5 rhythmic): ti, ab, de	65
5	rtms: ti, ab, de OR tms: ti, ab, de	22,778
6	((repetitive OR 'single pulse' OR 'paired pulse') NEAR/1 'transcranial magnetic stimulation'): ti, ab, de	8,395
7	#1 OR #2 OR #3 OR #4 OR #5 OR #6	33,851
8	'cocaine dependence'/exp	12,959
9	(cocaine* NEAR/2 (abuse* OR addict* OR dependen* OR disorder*)): ti, ab, de	17,794
10	#8 OR #9	17,794
11	#7 AND #10	124

WOS		
1	TS= (transcranial near/1 "magnetic stimulation*")<br>Índices=SCI-EXPANDED, SSCI, A&HCI, CPCI-S, CPCI-SSH, BKCI-S, BKCI-SSH, ESCI, CCR-EXPANDED, IC Período de tiempo=Todos los años	22,942
2	TS= (("transcranial magnetic stimulation" or tms) near/5 repetitive)<br>Índices=SCI-EXPANDED, SSCI, A&HCI, CPCI-S, CPCI-SSH, BKCI-S, BKCI-SSH, ESCI, CCR-EXPANDED, IC Período de tiempo=Todos los años	5,772
3	TS= (("transcranial magnetic stimulation" or tms) near/5 rhythmic)<br>*Índices=SCI-EXPANDED, SSCI, A&HCI, CPCI-S, CPCI-SSH, BKCI-S, BKCI-SSH, ESCI, CCR-EXPANDED, IC Período de tiempo=Todos los años*	41
4	TS= ((Repetitive or "single pulse" or "paired pulse") near/1 "transcranial magnetic stimulation")<br>Índices=SCI-EXPANDED, SSCI, A&HCI, CPCI-S, CPCI-SSH, BKCI-S, BKCI-SSH, ESCI, CCR-EXPANDED, IC Período de tiempo=Todos los años	6,106
5	#4 OR #3 OR #1<br>Índices=SCI-EXPANDED, SSCI, A&HCI, CPCI-S, CPCI-SSH, BKCI-S, BKCI-SSH, ESCI, CCR-EXPANDED, IC Período de tiempo=Todos los años	22,945
6	TS= (cocaine* near/2 (abuse* or addict* or dependen* or disorder*))<br>Índices=SCI-EXPANDED, SSCI, A&HCI, CPCI-S, CPCI-SSH, BKCI-S, BKCI-SSH, ESCI, CCR-EXPANDED, IC Período de tiempo=Todos los años	10,753
7	#6 AND #5<br>Índices=SCI-EXPANDED, SSCI, A&HCI, CPCI-S, CPCI-SSH, BKCI-S, BKCI-SSH, ESCI, CCR-EXPANDED, IC Período de tiempo=Todos los años	69

CINAHL		
S1	TI (transcranial n1 magnetic stimulation*) OR AB (transcranial n1 magnetic stimulation*)	
S2	TI (((transcranial magnetic stimulation or tms) n5 repetitive)) OR AB (((transcranial magnetic stimulation or tms) n5 repetitive))	
S3	TI (((transcranial magnetic stimulation or tms) n5 rhythmic)) OR AB (((transcranial magnetic stimulation or tms) n5 rhythmic))	
S4	TI ((rtms or tms)) OR AB ((rtms or tms))	
S5	TI (((Repetitive or "single pulse" or "paired pulse") n1 "transcranial magnetic stimulation")) OR AB (((Repetitive or "single pulse" or "paired pulse") n1 "transcranial magnetic stimulation"))	
S6	(MH "Transcranial Magnetic Stimulation")	
S7	S1 OR S2 OR S3 OR S4 OR S5 OR S6	3,752
S8	(MH "Substance Use Disorders")	37,484
S9	TI ((cocaine* n2 (abuse* or addict* or dependen* or disorder*))) OR AB ((cocaine* n2 (abuse* or addict* or dependen* or disorder*) S))	2,032
S10	S8 OR S9	38,553
S11	S7 AND S10	11

PsycINFO		
S1	TI (transcranial n1 magnetic stimulation*) OR AB (transcranial n1 magnetic stimulation*)	8,534
S2	TI (((transcranial magnetic stimulation or tms) n5 repetitive)) OR AB (((transcranial magnetic stimulation or tms) n5 repetitive))	3,242
S3	TI (((transcranial magnetic stimulation or tms) n5 rhythmic)) OR AB (((transcranial magnetic stimulation or tms) n5 rhythmic))	22
S4	TI ((rtms or tms)) OR AB ((rtms or tms))	7,707
S5	TI (((Repetitive or "single pulse" or "paired pulse") n1 "transcranial magnetic stimulation")) OR AB (((Repetitive or "single pulse" or "paired pulse") n1 "transcranial magnetic stimulation"))	3,457
S6	DE "Transcranial Magnetic Stimulation"	9,584
S7	S1 OR S2 OR S3 OR S4 OR S5 OR S6	11,859
S8	MM "Cocaine"	11,576
S9	TI ((cocaine* n2 (abuse* or addict* or dependen* or disorder*))) OR AB ((cocaine* n2 (abuse* or addict* or dependen* or disorder*)))	6,283
S10	S8 OR S9	13,565
s11	S7 AND S10	41

COCHRANE		
#1	MeSH descriptor: [Transcranial Magnetic Stimulation] explode all treES	1338
#2	(transcranial near/1 magnetic stimulation$)	4937
#3	((transcranial magnetic stimulation or tms) near/5 repetitive)	3268
#4	((transcranial magnetic stimulation or tms) near/5 rhythmic)	119
#5	(rtms or tms)	4596
#6	((Repetitive or "single pulse" or "paired pulse") near/1 "transcranial magnetic stimulation")	2891
#7	#1 or #2 or #3 or #4 or #5 or #6	6234
#8	MeSH descriptor: [Cocaine-Related Disorders] explode all trees	1037
#9	(cocaine* near/2 (abuse* or addict* or dependen* or disorder*))	2087
#10	#8 or #9	2087
#11	#7 and #10	39

Table S3. Ongoing studies about TMS for Cocaine use disorder.

Title	Status	Code	Estimated Study Completion Date
Treatment With Transcranial Magnetic Stimulation for Cocaine Addiction: Clinical Response and Functional Connectivity.	Recruiting	NCT02986438	December 31, 2020
Subjective Sleep Quality in CUD TMS-Treated	Recruiting	NCT03733821	December 2022
rTMS for Stimulant Use Disorders	Not yet recruiting	NCT04907357	December 2024
sTMS for Substance Use-disordered Veterans	Recruiting	NCT04336293	December 30, 2022
Theta-Burst Stimulation as a Treatment for Reducing Cocaine Use	Recruiting	NCT02927236	December 31, 2022
Neurocircuit Strategy to Decrease Cocaine Cue Reactivity	Recruiting	NCT04155632	December 31, 2024

Table S4. Quality assessment in nRCT.

**Sanna (2019)**	
**Domain**	**Rating**
Is it clear in the study what is the 'cause' and what is the 'effect' (i.e., there is no confusion about which variable comes first)?	Yes
Were the participants included in any comparisons similar?	Yes
Were the participants included in any comparisons receiving similar treatment/care, other than the exposure or intervention of interest?	Yes
Was there a control group?	Yes
Were there multiple measurements of the outcome both pre and post the intervention/exposure?	Unclear
Was follow up complete and if not, were differences between groups in terms of their follow up adequately described and analysed?	Yes
Were the outcomes of participants included in any comparisons measured in the same way?	Yes
Were outcomes measured in a reliable way?	Yes
Was appropriate statistical analysis used?	Yes
**Overall appraisal**	**High quality**

Table S5. Quality assessment in case-series.

Domain	Gómez (2020)	Madeo (2020)	Politi (2008)	Pettorruso (2019)	Rapinesi (2016)	Steele (2019)
**Study objective**						
Was the hypothesis/aim/objective of the study clearly stated?	Yes	Yes	Yes	Yes	Yes	Yes
**Study design**						
Was the study conducted prospectively?	Yes	Yes	Partially	Yes	Yes	Yes
Were the cases collected in more than one centre?	No	Unclear	Unclear	Unclear	No	Unclear
Were patients recruited consecutively?	Unclear	Unclear	Unclear	Unclear	Unclear	Unclear
**Study population**						
Were the characteristics of the patients included in the study described?	Yes	Yes	Partially	Yes	Yes	Yes
Were the eligibility criteria (i.e. inclusion and exclusion criteria) for entry into the study clearly stated?	Yes	No	Partially	Yes	Yes	Yes
Did patients enter the study at a similar point in the disease?	Unclear	Unclear	Unclear	Yes	Unclear	No
**Intervention and co-intervention**						
Was the intervention of interest clearly described?	Yes	Yes	Yes	Yes	Yes	Yes
Were additional interventions (co-interventions) clearly described?	No	No	No	No	No	No
**Outcome measure**						
Were relevant outcome measures established a priori?	Yes	Yes	Yes	Yes	Yes	Yes
Were the relevant outcomes measured using appropriate objective/subjective methods?	Yes	Yes	Yes	Yes	Yes	Yes
Were the relevant outcome measures made before and after the intervention?	Yes	Yes	Unclear	Yes	Yes	Yes
**Statistical analysis**						
Were the statistical tests used to assess the relevant outcomes appropriate?	Yes	Yes	Yes	Yes	Yes	Yes
**Results and conclusions**						
Was follow-up long enough for important events and outcomes to occur?	Yes	Yes	No	Yes	Yes	Yes
Were losses to follow-up reported?	Yes	Yes	No	Yes	Yes	Yes
Did the study provided estimates of random variability in the data analysis of relevant outcomes?	Yes	No	No	Yes	No	Yes
Were the adverse events reported?	Yes	Yes	Yes	Yes	Yes	Yes
Were the conclusions of the study supported by results?	Yes	Yes	Yes	Yes	Yes	Yes
**Competing interests and sources of support**						
Were both competing interests and sources of support for the study reported?	Yes	Yes	No	Yes	Yes	Partially
